# Smile aesthetics as perceived by dental students: a cross-sectional study

**DOI:** 10.1186/s12903-018-0673-5

**Published:** 2018-12-22

**Authors:** Juste Armalaite, Monika Jarutiene, Arunas Vasiliauskas, Antanas Sidlauskas, Vilma Svalkauskiene, Mantas Sidlauskas, Grazvydas Skarbalius

**Affiliations:** Clinic of Orthodontics, Lithuanian University of Health Sciences, Luksos-Daumanto st. 6, LT-50106 Kaunas, Lithuania

**Keywords:** Smile, Aesthetics, Dental education

## Abstract

**Background:**

An aesthetic smile has a number of components, and people generally equate a good dental appearance with success in many areas of life. The features that determine smile aesthetics could provide significant insights into post-treatment satisfaction and may predict a patient’s objectives when undergoing treatment. The purpose of this study was to evaluate how smile characteristics are perceived by dental students.

**Methods:**

The study was performed in 431 local and international dental students at the Lithuanian University of Health Sciences. The study data were collected using a three-part questionnaire. The first part of the questionnaire included sociodemographic items, i.e., student gender, age, nationality, and years of study; the second consisted of questions about facial aesthetic features; and the third elicited responses to photographs of 17 different smiles retrieved from the Lithuanian University of Health Sciences Clinic of Orthodontics database. The smile aesthetics were evaluated according to their dentolabial, dentogingival, dental, and dental arch characteristics using a 5-point numeric rating scale (1, best; 5, worst). The data were analysed using the Pearson’s chi-square and Mann-Whitney *U* tests.

**Results:**

The study included 336 local and 95 international dental students (132 men [30.6%], 299 women [69.4%]). Significantly more women than men focused on a person’s teeth when communicating (41.5% vs.32.6%, *p* < 0.005). Women were more critical than men when evaluating gingival smile, the ‘golden proportion’, occlusal cant, and dental crowding. The most unfavourable smile characteristics were identified in the dental analysis category, with hypodontia ranked as the worst smile feature (mean numeric rating scale score 4.71).

**Conclusion:**

Among dental students, the most distracting characteristics of a smile when determining its attractiveness were hypodontia, gingival smile, a reversed curvature of the occlusal plane, and dental crowding.

## Background

An increasing number of patients are seeking orthodontic treatment, usually because of concerns about their appearance. One of the main drivers of this high demand for aesthetic treatment is the influence of social media, especially among young adults [1]. Kiyak investigated the effects of orthodontic treatment on quality of life and reported that most patients who seek orthodontic treatment are wanting to enhance their aesthetic appearance and increase their chances of social acceptance rather than to improve their oral function or general health [2]. According to a meta-analytic review by Langlois et al., attractive children and adults receive more positive judgments and academic performance reviews than their less attractive counterparts, so are likely to have more self-confidence [3]. Moreover, malocclusion scores in children requiring orthodontic treatment have a negative psychosocial impact [4]. Although malocclusion is not a life-threatening condition, it is considered to be a public health problem because of its social implications. According to the World Health Organization, oral health is intertwined with general health, which in turn determines quality of life, and appropriate oral health care reduces mortality. Promoting and enhancing the overall health of patients by management of oral health is the primary goal of dentistry [5].

Smile aesthetics are defined by the teeth, which are framed by the lips, the contour of the gums, and the number of gaps and spaces. More precisely, the harmony and symmetry of an aesthetic smile is determined by the extent of exposure of the gingiva when smiling, the arc of the smile, the proportions of the teeth, the presence of a midline shift and changes in axial inclination, buccal corridors, gingival height and contours, presence of a diastema, and the colour of the teeth [6–10]. Although each factor may be considered individually, all components must act in concert to create an integrity that produces the final aesthetic effect. Furthermore, patients are becoming more critical of their smiles and are seeking orthodontic treatment with more specific expectations [11–13]. Accordingly, when making decisions about orthodontic treatment, it is crucial to understand the threshold of what the community considers acceptable in terms of smile features.

A pleasing smile is the result of an interaction of a number of components with varying degrees of importance, and an understanding of the principles that determine the balance between the knowledge of dental professionals regarding smile aesthetics and patients’ perceptions is essential. Competencies are defined as the abilities needed by the dental graduate to be able to embark on the practice of dentistry [14]. The General Assembly of the Association for Dental Education in Europe and the Dental Education Association in North America have identified the core and supporting dental competencies that graduate dental students should obtain, among which are the ability to identify a patient’s aesthetic requirements and to determine the degree to which those requirements or desires can be met [15, 16]. The smile often defines a person’s facial attractiveness, and so has a key role in social interaction. The correct order of priority of smile components when planning orthodontic treatment is a matter of debate [17–19]. The aims of this study were to identify the determinants of smile aesthetics as perceived by dental students and to examine factors that can alter the perception of smile characteristics.

## Methods

Four hundred and thirty-one local and international dental students at the Lithuanian University of Health Sciences were enrolled in this cross-sectional study, which was carried out between September 2012 and May 2016. A systematic random sampling technique was used to select the study sample from the registration lists of students admitted to each academic year. Year 4 and year 5 students were selected because of their more detailed knowledge of dentistry and smile aesthetics, and asked to complete a self-administered three-part questionnaire.

The study was reviewed and approved by the Kaunas Regional Biomedical Research Ethics Committee (approval number BE-2-12). A written informed consent form was read, understood, and signed by all the participants. In total, 453 questionnaires were distributed and 431 were returned; the remaining questionnaires were either not returned or were returned with one or more unanswered items. No respondent selection bias was identified and the sample was representative of the reference population.

**Fig. 1:**
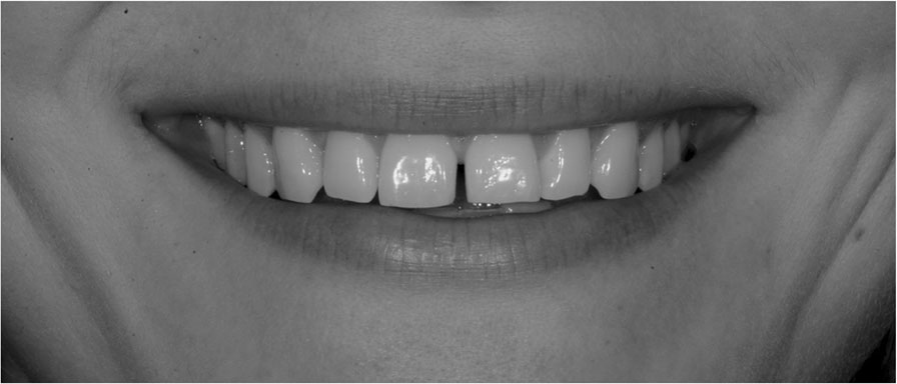
Example of the picture representing the feature of diastema

The first part of the questionnaire included sociodemographic items, i.e., gender, age, nationality, and years of study; the second part consisted of two close-ended questions about facial aesthetic features; and the third part elicited responses to photographs of the smiles from 17 different patients. These photographs were obtained when the patients attended their first visit at the Lithuanian University of Health Sciences Clinic of Orthodontics and were entered into the hospital’s Dolphin Imaging Software database. Several photographs were taken for each patient so that natural smiles could be obtained. The pictures were taken under the same environmental and lighting conditions and standardized using Adobe Photoshop (Adobe Systems, San Jose, CA, USA). The image chosen for the questionnaire was a frontal view showing the anterior teeth, the surrounding gingival tissues, and the lips (Fig. 1). The image was cropped to remove the chin, nose, and cheeks in order to minimise any confounding factors that could affect the perception of a smile [20]. The photographic inclusion criteria were that the images provided a frontal view, were of good quality, and represented only one dentolabial, dentogingival, dental, or dental arch smile characteristic, according to Fradeani diagnostic principles [21] (Table 1).

**Table 1:** Aesthetic variables examined

	Features	Description
Dentolabial analysis	Gingival smile	The amount of gingival shows above the central incisor crowns when smiling. More than 3mm is generally considered unattractive.
	Maxillary arch midline discrepancy	The relationship of the maxillary dental midline (measured between the central incisors) to the midline of the face, defined by the center of the philtrum. By definition, the ideal was considered to be 0 for this variable.
	Buccal corridor fill	The amount of dark space displayed between the facial surfaces of the posterior teeth and the corners of the mouth calculated as the total dark space on both sides of the mouth as a percentage of the total smile width. It can be absent (0 %), normal (1-13 %), wide (14-26 %).
Dentogingival analysis	Gingival margin discrepancy	The difference in the vertical height of the gingival zenith of the central incisor to the lateral incisor. The gingival margins of the maxillary central incisors and canines should be symmetric and in a more apical position compared to those of the lateral incisors.
	Hygiene	Poor oral hygiene (dental plaque on the teeth). Plaque is a yellow sticky film that forms on the teeth and gums and can be seen at gum margins of teeth with a food dye.
	Gum recession	The exposure in the roots of the teeth caused by a loss of gum tissue and/or retraction of the gingival margin from the crown of the teeth.
Dental analysis	Diastema	Open spaces between the upper incisors.
	Dental crowding	The dental arch length is less than the mesial distal width of the teeth intended to occupy it. Dental crowding occurs when the space required for the correct alignment of the teeth exceeds the space available in the dental arch. Crowding is classified to mild (2 – 3 mm), moderate (4 – 6 mm), severe (7 – 10 mm) and extreme (>10 mm).
	Protrusion of anterior teeth	Increased incisal profile in the anteroposterior direction. 2-3 mm is the normal horizontal overlap of the incisors.
	Incisor midline discrepancy	The relationship of the maxillary central embrasure to the mandibular central embrasure. By definition, the ideal was considered to be 0 for this variable.
	Hypodontia	A usually congenital condition of having fewer than the normal number of teeth. It is the developmental absence of 1 or more teeth.
	Anterior teeth colour	Abnormal tooth color, hue or translucency. External discoloration is accumulation of stains on the tooth surface. Internal discoloration is due to absorption of pigment particles into tooth structure.
	“Golden proportion”	A ratio of front teeth crown width and height. According to the golden proportion, the relationship between the maxillary central and lateral incisors and the canine should be as follows:1,62:1:0,62.
Dental arch analysis	Occlusal cant	The divergence of the occlusal plane from the horizontal axis, as seen when smiling. By definition, the ideal is considered to be 0° for this variable.
	Convex occlusal plane	Relationship between the curvature of the incisal edges of the maxillary anterior teeth and the curvature of upper border of the lower lip. As a rule, the incisal plane, when observed from the front, has a convex curve that follows the natural concavity of the lower lip during smiling.
	Flat occlusal plane	Relationship between the curvature of the incisal edges of the maxillary anterior teeth and the curvature of upper border of the lower lip is not parallel with flat maxillary incisal curvature to the upper border of lower lip.
	Reversed curvature of occlusal plane	Relationship between the curvature of the incisal edges of the maxillary anterior teeth and the curvature of upper border of the lower lip is not parallel with reverse maxillary incisal curvature to the upper border of lower lip.

**Fig. 2:**
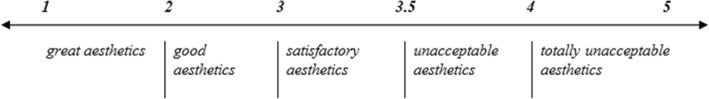
Numerical rating scale used to score smiles for their aesthetic value

The dental students were asked to evaluate each photograph aesthetically using a 5-point numeric rating scale (NRS; 1, best and 5, worst). The study was pretested for clarity in 30 dental students. From the pilot study, an NRS score ≥ 3.5 was chosen as the cut-off value for a smile that was no longer aesthetically acceptable (Fig. 2).

The statistical analysis was performed using IBM SPSS Statistics version 22.0 software (IBM Corp., Armonk, NY, USA). Descriptive statistics were used, i.e., the mean and standard deviation (SD) for frequency and the percentage for variables. The Mann-Whitney *U* test was used to compare differences in sociodemographic variables (e.g., gender and years of study) and in the dependent outcome variable (NRS scores for photographs) between groups. The Pearson’s chi-squared test was used to test the statistical significance of differences in responses according to sociodemographics (gender, years of study) and the answers to items in the second part of the questionnaire. A *p*-value of 0.05 was considered to be statistically significant.

The sample size was determined by a power analysis using G*Power version 3.1.9.2 software. Numeric rating scale was the variable for which we wanted an effect size of 0.4 as statistically significant when comparing men and women. We selected from a t- tests family with Mann-Whitney U (two groups) as a statistical test, two-tailed significance level (α) of 0.05 and a power of 0.9. Our calculation confirmed that the final sample size of 431 subjects was capable of identifying significant differences.

## Results

Of the 431 dental students who returned completed questionnaires, 336 were Lithuanian and 95 were from other countries; 256 (59.4%) were year 4 students and 175 (40.6%) were year 5 students. One hundred and thirty-two respondents (30.6%) were men, two hundred and ninety-nine (69.4%) were women. We did not test for an effect of respondent age because the majority (89.1%) of students were aged 20–25 years and only 10.9% were older. Similarly, we did not test for an effect of nationality because of the wide distribution of this demographic item.

The majority of respondents identified the smile as the most important facial aesthetic feature, and there was no statistically significant difference in response according to gender or years of study (Table 2). However, more women than men focused on a person’s teeth when communicating (41.5% vs. 32.6%, *p* < 0.005; Table 3), as did respondents with 5 years of training in dentistry when compared with those who had 4 years of training (41.8% vs.34.3%), but difference was not significant (*p* > 0.05).

**Table 2:** Distribution of responses to the question: “Do you think smile is one of the most important facial aesthetic features?”

Answer	Gender		Year of study	
	Male, n (%)	Female, n (%)	4th, n (%)	5th, n (%)
Yes	130 (98)	297 (99)	175 (100)	252 (98,5)
No	2 (2)	2 (1)	0 (0)	4 (1,5)
Total	132 (100)	299 (100)	175 (100)	256 (100)
*χ*^*2*^ statistics (df)	0.713 (1)		2.760 (1)	
p-value	0.398		0.097	

*df* Degrees of freedom

**Table 3:** Distribution of responses to the question: “Where are you looking at when communicating with people?”

Answer	Gender		Year of study	
	Male, n (%)	Female, n (%)	4th, n (%)	5th, n (%)
Eyes	36 (27.3)	44 (14.7)	34 (19.4)	46 (18.0)
Teeth	43 (32.6)	124 (41.5)	60 (34.3)	107 (41.8)
General appearance of face	53 (40.2)	131 (43.8)	81 (46.3)	103 (40.2)
Total	132 (100)	299(100)	175 (100)	256(100)
χ^2^ statistics (df)	9.937 (2)		2.524 (2)	
p-value	0.007		0.283	

*df* Degrees of freedom

**Fig. 3:**
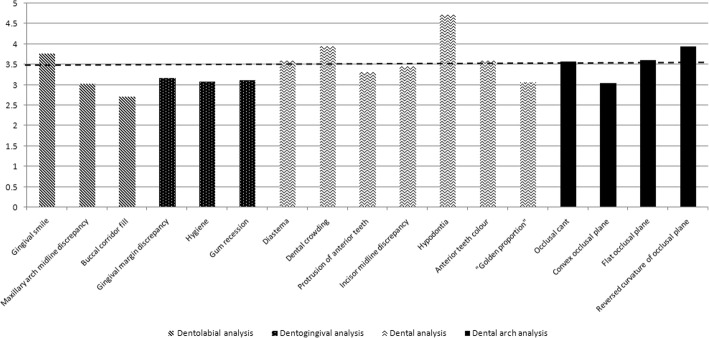
The mean values of smile features. - -- line representing the cut-off value for a smile that was no longer aesthetically acceptable (≥3.5)

The mean NRS scores for the photographs ranged from 2.72 (SD 0.66) to 4.71 (SD 0.67), with hypodontia being rated as the worst smile characteristic (Fig. 3). Women were more critical than men when evaluating the gingival smile, the ‘golden proportion’, occlusal cant, and dental crowding (Table 4). Years of study had no statistically significant impact on NRS ratings (p > 0.05, data not shown).

**Table 4:** Comparison of numeric rating scale scores for smile features according to student gender

Features	Mean numeric rating scale score (mean ± SD)		
	Men	Women	p*-*value^a^
Gingival smile	3.55 ± 0.52	3.87 ± 0.53	0.001^b^
Maxillary archmidline discrepancy	3.03 ± 0.86	3.11 ± 0.49	0.283
Buccal corridor fill	2.72 ± 0.66	2.89 ± 0.69	0.077
Gingival margin discrepancy	3.17 ± 0.89	3.05 ± 0.79	0.171
Hygiene	3.09 ± 0.75	3.21 ± 0.46	0.193
Gum recession	3.11 ± 0.84	3.27 ± 0.78	0.198
Diastema	3.60 ± 0.80	3.51 ± 0.84	0.283
Dental crowding	3.79 ± 0.61	4.00 ± 0.59	0.004^b^
Protrusion of anterior teeth	3.31 ± 0.73	3.32 ± 0.81	0.948
Incisor midline discrepancy	3.45 ± 0.88	3.58 ± 0.49	0.117
Hypodontia	4.71 ± 0.67	4.79 ± 0.59	0,082
Anterior teeth colour	3.59 ± 0.82	3.63 ± 0.57	0.592
“Golden proportion”	3.11 ± 0.63	3.34 ± 0.42	0.01^b^
Occlusal cant	3.41 ± 0.78	3.64 ± 0.75	0.006^b^
Convex occlusal plane	3.04 ± 0.83	3.16 ± 0.78	0.188
Flat occlusal plane	3.57 ± 0.84	3.54 ± 0.71	0.943
Reversed curvature of occlusal plane	3.95 ± 0.84	3.97 ± 0.74	0.55

^a^Mann-Whitney U test

^b^statistically significant difference

## Discussion

It is believed that attractive people are more likely to obtain better jobs, have more successful marriages, and experience happier, more fulfilling lives. These societal expectations start early in childhood and last a lifetime. Poor dental aesthetics have been linked to lack of self-confidence and are thought to be socially, academically, and occupationally disadvantageous [19]. Younger generations are attaching increasing importance to all aspects of their appearance and the role of an attractive smile is undeniable [12]. Women tend to be more interested in their appearance than men, and female patients have been reported to be more concerned with their dental appearance than male patients and to be more critical when judging smile aesthetics [11, 22, 23]. Ong et al. reported that dental attractiveness was rated as being more important by women than by men [24]; however, this finding was not confirmed in other studies [25, 26]. In our study, female students were significantly more critical than men when evaluating gingival smile, the “golden proportion”, dental crowding, and occlusal cant. An earlier study in Lithuania showed that girls were more likely than boys to complain about malocclusion (53.6% vs. 41.8%) [27]. According to Josefsson et al., girls aged 18–19 years perceived a greater need for orthodontic treatment of their malocclusion than their male counterparts [28]. In contrast, Hamdanet al. found no statistically significant gender-related difference in the cut-off point determining the desire for orthodontic treatment [29]. According to Manipal et al., 42.5% of female students and 69.2% of male students believed that their dental appearance hindered their careers, and 95.4 and 92.3%, respectively, were aware of dental aesthetics [30]. Similarly, the majority (98.3%) of respondents in our study accepted that the smile is one of the main aesthetic features of the face. In a study by Abidia et al.*,* the majority (89.4%) of female students considered that their teeth determined their facial attractiveness; almost one third (30.8%) reported trying to hide their smile, nearly one half (51%) were not satisfied with the colour of their teeth, and around two thirds (61.5%) felt that their quality of life was affected by their dental appearance [31].

Dental students are part of the dental workforce and should be able to identify patients’ needs and expectations, make clinical decisions pertaining to dental aesthetics, and know when to intervene or refer. The literature contains little information regarding the perception of smile aesthetics on the part of dental students as compared with laypersons. Our present study represents only an early step in research on smile aesthetics. Year 4 and year 5 students can be regarded as dental professionals and year 1 and year 2 students as laypersons for the purpose of studying how the perception of smile aesthetics changes during the years of professional training. Dental students in their clinical years should be encouraged to discuss the differences in perception of smile aesthetics between professionals and laypeople when planning treatment with patients. Understanding this difference in perception is important to be able to address the patient’s needs and expectations in regard to aesthetics [20].

Cracel-Nogueira and Pinho compared the aesthetic perception of several components of the smile by laypersons, dental students, and dental professionals, and found that all had different perceptions of attractiveness when evaluating several modified smile features. A smile with minimal exposure of the gingiva (≤2 mm) was the most appreciated, whereas a gingival smile (≥3 mm) and presence of a diastema were considered to be the least aesthetically pleasing. A midline shift was the least perceptible aesthetic feature. Laypersons tended to be more tolerant than professionals when evaluating smile characteristics [32]. Kokich et al. also compared the perceptions of dental professionals with those of laypeople and, like Cracel-Nogueira and Pinho, found that both groups agreed that 3 mm of gingival display resulted in an unattractive smile [18]. In our study, the gingival display was 3 mm, which was rated as aesthetically unattractive by dental students, as was the presence of a diastema. Other researchers compared the perception of smile aesthetics between dental and non-dental students and found that both groups were less forgiving of dark tooth shades [20] but found no difference in the perception of a diastema. Those authors also found that both dental and pharmacy students were sensitive to a midline shift of 2 mm [20] and reported that their findings were similar to those of another study that found a midline shift of 2 mm was perceived by 83% of orthodontists and dental professionals and by 56% of laypersons [33]. In our study, a shift in the midline of the maxillary arch was rated as aesthetically acceptable. According to Cardash et al., the threshold for a midline discrepancy is ≥2 mm [7]. Ker et al. established the acceptable value to be 2.9 mm [13]. We conclude that a small discrepancy in the midline of the maxillary arch is not noticeable and could remain uncorrected if it is not of concern to the patient.

This study has several limitations that need to be taken into account when interpreting its results. First, we used only one of several existing approaches to evaluate smile aesthetics. Use of other instruments and objective clinical examinations would have created the opportunity for similar studies in the future. The findings of studies of the perception of smile aesthetics reported to date have varied widely in terms of analytical methods and data collection instruments (web-based surveys, self-reported perception, photographs, software-altered images) and included a wide range of smile features and sociocultural parameters, so it is difficult to compare their findings. Another limitation is that we used images only with the features of smile aesthetics that we considered important and wanted to evaluate. However, there are no internationally recognised standardised photographs for rating smile aesthetics or relevant studies, so we were unable to undertake an objective comparison of our findings with those of other investigators. A more comprehensive questionnaire and inclusion of clinical examinations would have been helpful, but was not possible in view of limited funding resources. Another limitation was that the subjects were in different study years and had different teachers, who may have influenced their perception of smile aesthetics. However, the students were from the same university and were undertaking the same programme of study.

## Conclusions

Among dental students, the most distracting characteristics when assessing smile attractiveness were hypodontia, gingival smile, a reverse occlusal plane, and dental crowding. Women were more critical than men when evaluating a gingival smile, the ‘golden proportion’, occlusal cant, and dental crowding.

## Abbreviations

NRS: Numeric rating scale
SD: Standard deviation
